# Extrarenal Papillary Renal Cell Carcinoma: A Unique Case With Bilateral Involvement of the Adrenal Glands

**DOI:** 10.1155/criu/9413935

**Published:** 2026-11-06

**Authors:** Margarida André, Brigitta Cismasiu, Luísa Moreira, Marta Vasconcelos, Maria Isabel Santos, João Rodrigues-Alves, Eduardo Fernandes, João Paulo Rosa, Miguel Carvalho, Helder Mansinho, Carlos Luz-Correia

**Affiliations:** ^1^ Urology Department Almada-Seixal Local Health Unit Garcia de Orta Hospital Almada Portugal hgo.pt; ^2^ Surgery Department Almada-Seixal Local Health Unit Garcia de Orta Hospital Almada Portugal hgo.pt; ^3^ Pathology Department Almada-Seixal Local Health Unit Garcia de Orta Hospital Almada Portugal hgo.pt; ^4^ Radiology Department Almada-Seixal Local Health Unit Garcia de Orta Hospital Almada Portugal hgo.pt; ^5^ Medical Oncology Department Almada-Seixal Local Health Unit Garcia de Orta Hospital Almada Portugal hgo.pt

**Keywords:** adrenal gland, extrarenal renal cell carcinoma, papillary renal cell carcinoma

## Abstract

Primary extrarenal renal cell carcinoma (RCC) is an exceptionally rare malignancy. We report a unique case of bilateral adrenal papillary extrarenal RCC in a 62‐year‐old man, managed through sequential surgical interventions over a prolonged follow‐up. Initial right adrenalectomy revealed a papillary RCC with high‐grade oncocytic features; immunohistochemical analysis confirmed PAX8 positivity, supporting a renal‐type origin in the absence of a renal primary. Subsequent contralateral adrenal involvement and local recurrence required laparoscopic adrenalectomy and multiorgan resection. Despite complete macroscopic resections, the disease progressed to hepatic, pulmonary, peritoneal, and cerebral metastases, resulting in the patient′s death due to postoperative intracranial hemorrhage. No renal involvement was identified throughout the disease course. This case highlights the aggressive behavior of extrarenal RCC and underscores the importance of comprehensive immunohistochemical workup, prolonged surveillance, and multidisciplinary management in the absence of standardized guidelines.

## 1. Introduction

Renal cell carcinoma (RCC) accounts for more than 90% of primary kidney neoplasms. Clear cell RCC is the most frequent histological subtype (75%–80%), followed by papillary RCC, which represents approximately 10%–15% of cases [1, 2].

Extrarenal RCC is an exceptionally rare entity, described only sporadically in the literature. It is defined as the presence of RCC arising in extrarenal tissue in the absence of any detectable renal lesion [3]. Data regarding its incidence, pathogenesis, diagnoses, optimal management, and follow‐up are scarce. We present a case of extrarenal papillary RCC with bilateral adrenal involvement, reported in accordance with the CARE guidelines.

## 2. Clinical Case

In 2016, a 62‐year‐old man with a medical history of hypertension, gastroesophageal reflux disease, and former tobacco use was referred to the outpatient clinic, following the incidental detection of a right adrenal mass on routine abdominal ultrasound. Further evaluation with contrast‐enhanced abdominal computed tomography (CT) and magnetic resonance imaging (MRI) revealed a 7.7 × 4.1 × 4.9 − cm bilobed right adrenal nodule near the inferior vena cava, without evidence of renal lesions or adenopathies (Figure 1). Dedicated assessment of both kidneys on CT and MRI demonstrated normal renal morphology, with no solid lesion, complex cyst, or parenchymal scar to suggest a current or previously regressed renal tumor.

**Figure 1:**
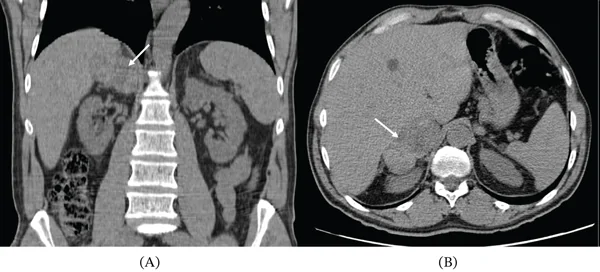
(A) Coronal abdominal CT demonstrating a 7.7 × 4.1 × 4.9 − cm bilobed right adrenal mass (white arrow) adjacent to the inferior vena cava. (B) Corresponding axial abdominal CT of the same lesion (white arrow). Both kidneys are morphologically normal, with no renal lesion or adenopathy identified.

The patient underwent right adrenalectomy through a median laparotomy. Histopathological examination demonstrated a 7.5‐cm papillary RCC with high‐grade oncocytic features, ISUP Grade 3, with negative surgical margins. Immunohistochemical analysis showed tumor cells positive for PAX8, RCC marker, CD10, racemase, CK7 (focal), and epithelial membrane antigen (EMA) (focal), and negative for CK20, chromogranin A, synaptophysin, inhibin, melan‐A, CD117, calretinin, WT1, TTF‐1, and napsin‐A. Fumarate hydratase (FH) expression was retained, and TFE3 immunostaining was negative (Figure 2).

**Figure 2:**
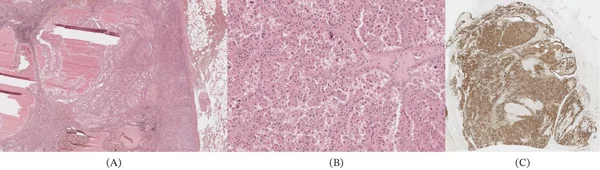
(A) H&E, ×40: well‐circumscribed papillary tumor adjacent to adrenal cortical parenchyma. (B) H&E, ×200: papillae with fibrovascular cores lined by cells showing nuclear pseudostratification, round‐to‐oval nuclei with irregular nuclear membranes, prominent nucleoli, and abundant eosinophilic cytoplasm. (C) Immunohistochemistry: positive staining for racemase, with similar immunoreactivity for RCC marker and CD10.

Three months after surgery, follow‐up MRI identified a 10 × 6 − mm nodule in the left adrenal gland, with both kidneys remaining morphologically normal. A positron emission tomography (PET) scan, thoracic CT, and cerebral MRI were subsequently performed and showed no additional suspicious lesions; therefore, a strategy of clinical surveillance was adopted.

At 1 year of follow‐up, interval growth of the left adrenal lesion was observed, measuring 14 × 11 mm (Figure 3). Further investigation with endoscopic ultrasound‐guided biopsy was performed, and histological analysis revealed features consistent with papillary RCC, similar to the initial tumor. The patient subsequently underwent retroperitoneal laparoscopic left adrenalectomy, and pathological examination confirmed contralateral adrenal involvement by morphologically similar papillary RCC.

**Figure 3:**
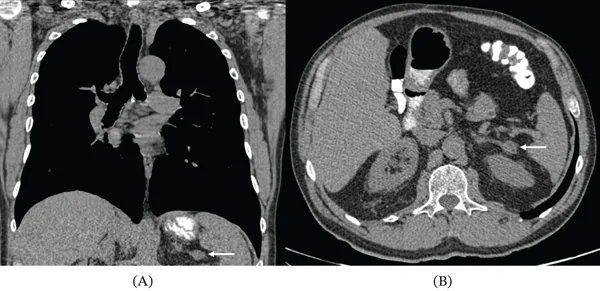
(A) Coronal abdominal CT demonstrating a 14 × 11 − mm left adrenal mass (white arrow). (B) Corresponding axial abdominal CT of the same lesion (white arrow). The left kidney is morphologically normal.

One year later, surveillance CT demonstrated local tumor recurrence (Figure 4), with invasion of the splenic artery and pancreatic tail. The patient underwent multiorgan resection through a median laparotomy. Pathological analysis confirmed locally recurrent papillary RCC with negative margins. In the absence of radiological or pathological evidence of residual disease, no adjuvant therapy was initiated.

**Figure 4:**
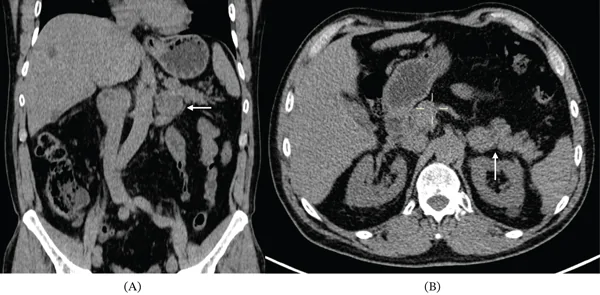
(A) Coronal abdominal CT demonstrating local tumor recurrence following left adrenalectomy (white arrow) invading the splenic artery and pancreatic tail. (B) Corresponding axial abdominal CT showing the same recurrence (white arrow).

The patient continued follow‐up with MRI every 6 months and annual PET imaging until the fourth year after initial diagnosis (2020), when hepatic, pulmonary, and peritoneal metastases were detected. Palliative systemic therapy with sunitinib was initiated. One month later, the patient was admitted to the emergency department with aphasia and headache. Brain imaging revealed multiple metastatic lesions, the largest located in the left temporal lobe. Craniotomy with surgical excision of the dominant lesion was performed; however, the patient died in the postoperative period due to intracranial hemorrhage. Throughout the entire disease course, spanning 4 years of imaging surveillance with CT, MRI, and PET, no renal lesions were identified.

## 3. Discussion

Extrarenal RCC is a remarkably rare and still poorly understood entity, defined by the presence of a renal‐type carcinoma arising outside the kidney in the absence of a detectable renal primary tumor [3, 4]. Since its initial descriptions, only a limited number of cases have been reported, most as isolated case reports or very small series, reflecting both its rarity and the diagnostic challenges it poses [3–7].

Clear cell RCC is the most frequently reported histological subtype in extrarenal locations; papillary RCC with high‐grade oncocytic features is very rare [3, 6, 7]. To date, only a handful of cases of primary extrarenal papillary RCC have been described, with variable anatomical locations including the retroperitoneum, adrenal glands, pelvis, and lymphatic tissue [3, 6, 7]. The principal published cases of primary extrarenal RCC are summarized and compared with the present case in Table 1. Notably, the few reported adrenal or periadrenal cases have almost invariably been of clear cell morphology [9, 10], and bilateral adrenal involvement, as observed in our patient, is exceptional; to our knowledge it has not been previously described with such a prolonged and well‐documented clinical course.

**Table 1:** Reported cases of primary extrarenal renal cell carcinoma compared with the present case.

Study	Age/sex	Site	Histology	Treatment	Outcome
Terada [5]	83/M	Near lower pole of right kidney	Clear cell	Complete excision	No recurrence at 6 months
Li et al. [3]	63/M	Retroperitoneum, lateral to IVC	Papillary, Type 2	Complete excision	No recurrence at 5 months
AlShalabi et al. [8]	62/F	Perirenal, heterotopic renal anlage	Clear cell	Complete excision	Death, 3 months after surgery
Srivishnu et al. [6]	34/M	Neck mass; para‐aortic mass	Papillary, Type 2	Tyrosine kinase inhibitor, palliative	Response not assessed
Mansoor et al. [4]	54/M	Left perirenal region	Clear cell	Complete excision	No recurrence at 8 months
	77/M	Right adrenal	Unclassified extrarenal RCC	Complete excision	No recurrence at 20 months
	63/F	Retroperitoneal lymphadenopathy	Clear cell	Complete excision	No recurrence at 16 months
Sarafi et al. [7]	57/F	Pelvis	Papillary, Type 2	Surgical excision	No recurrence at 6 months
Yao et al. [9]	48/F	Left adrenal region	Clear cell	Complete excision	No recurrence at 6 months
Alayed et al. [10]	15/F	Left adrenal and pulmonary lesion	TFE3 translocation‐associated	Left lower lung lobe resection, followed by nivolumab and adrenal resection	No recurrence at 12 months
Present case	62/M	Bilateral adrenal	Papillary, high‐grade oncocytic, ISUP 3	Sequential resections; sunitinib	Death, 4 years after diagnosis

*Note:* Papillary subtypes are reported as stated in the original publications.

Abbreviations: ISUP, International Society of Urological Pathology; IVC, inferior vena cava; RCC, renal cell carcinoma.

The clinical presentation of extrarenal RCC is heterogeneous and largely dependent on tumor location. Many cases are discovered incidentally during imaging performed for unrelated reasons, particularly when located in the adrenal or retroperitoneal region [9, 11]. Imaging typically demonstrates a solid enhancing mass without renal involvement; however, radiological findings alone are insufficient to establish the diagnosis, necessitating histopathological and immunohistochemical confirmation [3, 4]. In the adrenal location, PAX8 expression is a critical marker for establishing renal‐type differentiation, and systematic exclusion of adrenocortical, neuroendocrine, mesothelial, pulmonary, and thyroid primaries through an extended immunohistochemical panel is required before a diagnosis of primary extrarenal RCC can be confidently rendered [4, 12, 13].

A diagnosis of primary extrarenal RCC requires the systematic exclusion of three groups of alternatives: an occult or regressed renal primary, nonrenal tumors with papillary architecture, and molecularly defined renal neoplasms that mimic papillary RCC morphologically. In our patient, an occult renal primary was the principal differential consideration. It was excluded on the basis of repeated dedicated imaging of both kidneys —contrast‐enhanced CT and MRI at diagnosis and at each surveillance interval, together with annual PET—over 4 years, none of which demonstrated a renal mass, a complex cyst, or a parenchymal scar suggestive of a spontaneously regressed tumor. Furthermore, no residual normal renal tissue was identified within any of the resected specimens, arguing against a supernumerary or ectopic kidney [4, 5]. Nonrenal primaries were excluded immunohistochemically: adrenocortical carcinoma with oncocytic features by negativity for inhibin, melan‐A, and calretinin in the presence of diffuse PAX8 expression, which adrenocortical tumors characteristically lack [12]; neuroendocrine neoplasia by negativity for chromogranin A and synaptophysin; malignant mesothelioma by negativity for calretinin and WT1; and metastatic pulmonary adenocarcinoma by negativity for TTF‐1 and napsin‐A. Metastatic thyroid and Müllerian carcinomas, which may also express PAX8 [13], were considered improbable given the male sex of the patient, the negative TTF‐1 staining, and the absence of any thyroid or pelvic lesion on PET and cross‐sectional imaging. Finally, the molecularly defined mimics of high‐grade papillary morphology were addressed directly: Retained FH expression excluded FH‐deficient RCC, and negative TFE3 immunostaining did not support a diagnosis of MiT family translocation RCC—an entity that has itself been reported in an extrarenal adrenal location [10]. We acknowledge, however, that TFE3 immunohistochemistry has lower sensitivity than fluorescence in situ hybridization, and that molecular confirmation was not performed; this represents a limitation of our diagnostic workup.

Several hypotheses have been proposed to explain the pathogenesis of extrarenal RCC. One theory suggests malignant transformation of embryonic mesonephric or metanephric remnants persisting outside the kidney, which could justify the frequent proximity of these tumors to renal or adrenal structures [5]. Another hypothesis postulates the possibility of an occult or regressed primary renal tumor; however, long‐term imaging follow‐up without evidence of renal lesions, as in our case, argues against this explanation [4, 5]. Importantly, none of the reported cases, including ours, identified residual normal renal tissue within the tumor specimen, supporting the concept of a true extrarenal origin [4, 5].

Management strategies for extrarenal RCC are not standardized due to the scarcity of cases. Complete surgical excision with negative margins remains the treatment of choice in localized disease and has been associated with favorable short‐term outcomes in most reported cases [3, 6, 7, 9, 11]. Nevertheless, the long‐term behavior of these tumors remains unpredictable. Although several reports describe indolent courses without recurrence, aggressive progression with local recurrence and distant metastases has also been documented [7, 8].

Systemic therapy in this patient was initiated in 2020, when sunitinib was the guideline‐endorsed first‐line standard of care for metastatic papillary RCC, in the absence of any randomized evidence supporting an alternative agent. The therapeutic landscape has since changed substantially. The Randomized Phase 2 PAPMET trial, first presented and published in February 2021, demonstrated significantly longer progression‐free survival with cabozantinib than with sunitinib in metastatic papillary RCC (hazard ratio 0.60, 95% CI 0.37–0.97) [14]. Subsequently, immune checkpoint inhibitor combinations have shown encouraging activity in nonclear‐cell disease. In the single‐arm Phase 2 KEYNOTE‐B61 trial of lenvatinib plus pembrolizumab, the objective response rate was 54% in the papillary subgroup at a median follow‐up of 15 months [15]. In a Phase 2 trial of cabozantinib plus nivolumab in 40 patients with papillary, unclassified or translocation‐associated RCC, the objective response rate was 48% overall and 47% among the 32 patients with papillary histology, with a median progression‐free survival of 13 months (95% CI 7–16) at a median survivor follow‐up of 34 months [16]. The randomized SUNNIFORECAST trial compared nivolumab plus ipilimumab with investigator′s choice of standard of care in 309 treatment‐naive patients with advanced nonclear‐cell RCC, of whom 56% had papillary histology on central pathology review. It met its primary endpoint of the 12‐month overall survival rate (78%, 95% CI 71%–84%, vs. 68%, 95% CI 60%–75%; *p* = 0.026); median overall survival was 33.2 versus 25.2 months (HR 0.81, 95% CI 0.61–1.09) and median progression‐free survival did not differ between arms (5.4 vs. 5.7 months). In the papillary subgroup, median overall survival was 28.4 versus 18.9 months (HR 0.84, 95% CI 0.59–1.21), and no major difference between papillary and nonpapillary subtypes was observed for any endpoint [17]. Accordingly, the 2026 European Association of Urology guidelines recommend cabozantinib for patients with papillary RCC on the basis of a positive randomized controlled trial, and lenvatinib plus pembrolizumab or nivolumab plus cabozantinib on the basis of small single‐arm trials; nivolumab plus ipilimumab is recommended for nonclear‐cell RCC more broadly. All of these are weak‐strength recommendations, and the panel advises that patients with nonclear‐cell metastatic RCC be referred to a clinical trial where appropriate [1]. It is important to emphasize that none of these trials enrolled a patient with primary extrarenal RCC, and whether their conclusions extend to this entity is entirely unknown. It should also be noted that our patient′s clinical course was unusually compressed: Cerebral metastases became symptomatic approximately 1 month after sunitinib was started, and death followed shortly thereafter, so that no meaningful response assessment was possible for any systemic agent. Were a comparable patient to present today, an immune checkpoint inhibitor–based combination or cabozantinib would be considered within a multidisciplinary framework, with clinical trial enrollment favored wherever available.

Our case illustrates an aggressive disease trajectory, characterized by contralateral adrenal involvement, local recurrence, systemic dissemination, and fatal outcome despite repeated complete surgical resections and prolonged surveillance. The exceptionally long follow‐up period highlights that extrarenal RCC with high‐grade oncocytic papillary morphology may behave similarly to high‐risk renal RCC, with late recurrences and metastatic spread [1, 7, 8]. The absence of established recommendations regarding adjuvant therapy or optimal surveillance protocols further complicates clinical decision‐making. In our case, all therapeutic decisions were made within a multidisciplinary oncologic framework, reflecting current best practice in the management of rare malignancies.

This case adds valuable longitudinal data to the limited existing literature and underscores the need for heightened clinical awareness, meticulous long‐term follow‐up, and further reporting of similar cases to better characterize the natural history and optimal management of extrarenal RCC.

## 4. Conclusions

Primary extrarenal papillary RCC is an extremely rare entity with uncertain pathogenesis and unpredictable clinical behavior. This case represents a unique presentation with bilateral adrenal involvement, local recurrence, late metastatic progression, and one of the longest follow‐up periods reported to date. Complete surgical resection remains the mainstay of treatment; however, the risk of late recurrence and metastasis warrants prolonged surveillance. Further accumulation of well‐documented cases is essential to improve understanding of disease biology and to develop evidence‐based recommendations for follow‐up and adjuvant treatment.

**Nomenclature**
AMACR: alpha‐methylacyl‐CoA racemase (also referred to as racemase or P504S)
CK7: Cytokeratin 7
CK20: Cytokeratin 20
CT: computed tomography
EMA: epithelial membrane antigen
FH: fumarate hydratase
H&E: hematoxylin and eosin
IHC: immunohistochemistry
ISUP: International Society of Urological Pathology (grading system)
MiT: microphthalmia transcription factor family
MRI: magnetic resonance imaging
PAX8: paired box gene 8 transcription factor
PET: positron emission tomography
RCC: renal cell carcinoma
TFE3: transcription factor E3 (MiT family translocation marker)
TTF‐1: thyroid transcription factor 1
WHO: World Health Organization
WT1: Wilms tumor protein 1

## Author Contributions

Margarida André: conceptualization, investigation, writing—original draft, and writing—review and editing. Brigitta Cismasiu: investigation and writing—original draft. Luísa Moreira: investigation and writing—original draft. Marta Vasconcelos: investigation and writing—original draft. Maria Isabel Santos: investigation and writing—original draft. João Rodrigues Alves: histopathological evaluation, formal analysis, and writing—review and editing. Eduardo Fernandes: radiological analysis and interpretation, formal analysis, and writing—review and editing. João Paulo Rosa: supervision and writing—review and editing. Miguel Carvalho: supervision and writing—review and editing. Helder Mansinho: oncological clinical follow‐up and writing—review and editing. Carlos Luz‐Correia: surgical management and clinical follow‐up, and writing—review and editing.

## Funding

No funding was received for this manuscript.

## Disclosure

All authors have read and approved the final version of the manuscript. Margarida André had full access to all of the data in this study and takes complete responsibility for the integrity of the data and the accuracy of the data analysis.

## Ethics Statement

Ethical review and approval were waived for this study, as it constitutes a single case report with no experimental intervention. The study was conducted in accordance with the Declaration of Helsinki. The patient died during the reported episode of care; written informed consent for publication of this case, including all clinical images, was subsequently obtained from the patient′s next of kin. All potentially identifying information has been removed from the manuscript and figures.

## Conflicts of Interest

The authors declare no conflicts of interest.

## Supporting information


**Supporting Information** Additional supporting information can be found online in the Supporting Information section. 

## Data Availability

Data sharing is not applicable to this article as no datasets were generated or analysed during the current study.
